# Relationship Between Lumbar Motor Control Ability and Spinal Curvature in Elderly Individuals

**DOI:** 10.3390/healthcare8020130

**Published:** 2020-05-09

**Authors:** Ryo Miyachi, Junya Miyazaki

**Affiliations:** Department of Physical Therapy Faculty of Health Science, Kyoto Tachibana University, Kyoto 607-8175, Japan; j-miyazaki@tachibana-u.ac.jp

**Keywords:** motor control, movement pattern, spinal curvatures, lumbar, prone hip extension

## Abstract

This study aimed to clarify the relationship between spine curvature and the movement pattern/motor control ability of the lumbar and hip joints during prone hip extension in elderly individuals. The participants were 14 elderly people who attended a community health class. We measured the motion angle, motion ratio (movement pattern), and motor control ability of the lumbar and hip joints during prone hip extension. In addition, the lumbar lordosis angle and thoracic kyphosis angle were measured in the standing position. There was no correlation between the spinal curvature in the standing position and the lumbar/hip joint movement pattern and motor control ability during prone hip extension. When evaluating the lumbar spine, it is necessary to perform a comprehensive evaluation by interpreting static evaluations such as spinal alignment or dynamic evaluations such as movement patterns and motor control abilities.

## 1. Introduction

Repetitive or sustained loading due to habitual movement patterns in daily life leads to tissue microinstability and microinjury. In addition, excessive loading is applied to joints and tissues with high relative flexibility [1]. Regarding the relationship between the lumbar and the hip joints, it is said that excessive lumbar motion tends to appear when the hip joint is moved because of the difference in relative flexibility, and the load is added to the lumbar spine [1]. People with low back pain have early lumbar movement when they move their limbs [2], and excessive lumbar movement occurs in their daily lives [3]. Furthermore, individuals with low back pain have a lower ability to correct excessive movement of the lumbar spine than those without low back pain [4]. In other words, people with low back pain are considered to have a pattern of excessive movement of the lumbar spine and lack the ability to control excessive movement of the lumbar spine when they move their lower limbs. There are many reports on the movement patterns and motor control of the lumbar spine during lower limb movements. In addition, these reports have been verified in various movements such as knee extension in the sitting position, active straight leg raise, and hip abduction/external rotation in the clock lying position [5,6,7,8,9,10]. In particular, prone hip extension (PHE) is often used not only as an assessment of the hip extensor muscle strength but also as a test to assess lumbar movement patterns and motor control abilities [11]. Although there are no reports of intra-rater reliability of PHE, the inter-rater reliability of PHE has been presumed to be good [12,13]. In addition, there is an association between lumbar motor control ability and lower back pain during PHE [11,12]. In contrast, it is widely known that the body adapts to habits that have a specific direction of movement [14,15]. The evaluation of spinal alignment is one of the most frequently performed evaluations and is used not only in relation to various physical functions [16] but also as a predictor of the movement patterns frequently used in daily life and the manner in which the load is applied to the spine. Although inconsistent, associations between spinal alignment and low back disease and low back pain have been reported [17,18,19,20,21]. If a movement pattern of hyperextension of the lumbar spine during PHE is observed, it is expected that a tendency to routinely hyperextend the lumbar or adaptive changes resulting from this tendency may affect the spinal alignment. In particular, elderly individuals are more likely to adopt a particular posture due to the influence of longstanding habits in a particular direction of movement compared to the young and are likely to have structural changes in the alignment of the spine. In considering the load on the lumbar spine, a comprehensive approach is taken by combining the evaluation of movement patterns, motor control ability, and spinal alignment of the lumbar spine; however, the relationship between them has not been clarified. This study aimed to clarify the relationship between spinal curvatures, movement patterns, and motor control ability of the lumbar and hip joints during PHE. 

## 2. Materials and Methods 

### 2.1. Participants

A total of 14 elderly people, 3 males (age: 74.3 ± 3.8 years, height 162.3 ± 4.1 cm, weight 63.4 ± 6.2 kg) and 11 females (age: 72.4 ± 1.3 years, height 151.3 ± 1.2 cm, weight 50.5 ± 1.1 kg) attending a community health class were included. After explaining the aim and methods of the study orally and in writing, those who agreed to provide written consent to participate in the study “voluntarily” were considered. The participants were elderly individuals who were independent enough to participate in the study by themselves. Participants with dementia, those who did not fully understand the purpose and method of the study, those who were certified as requiring nursing care, those who complained of severe pain that interfered with daily life, those with typical physical disabilities such as cerebrovascular disease and rheumatoid arthritis, and those whose range of motion of hip joint extension was less than 0° were excluded.

The study was conducted in accordance with the Declaration of Helsinki, and the protocol was approved by the Ethics Committee of Kyototatibana University (approval number: 19-30).

### 2.2. Assessment of Movement Patterns and Motor Control Abilities During PHE

**Figure 1:**
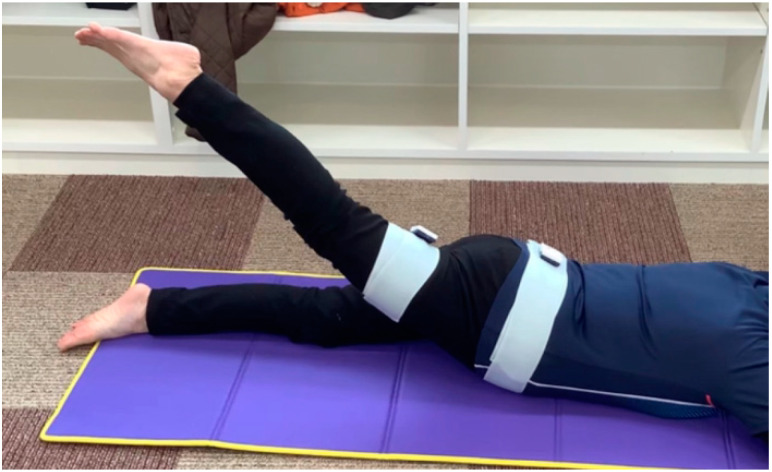
Measurement of pelvic and femoral movement during prone hip extension (PHE) using an inertial sensor.

To investigate the movement pattern and motor control ability of the lumbar, pelvic, and femoral regions, the movements during PHE were measured using an inertial sensor (TSND151, ATR-Promotions) and receiving software (Sensor Controller, ATR-Promotions). The inertial sensors were attached to the pelvis and right femoral region. The pelvic sensor was placed at the center of the sacrum, where the upper edge of the sensor was the line connecting the superior posterior iliac spine on both sides. The femoral sensor was placed on the posterior surface of the femoral region, midway between the sciatic tubercle and fossa poplitea. The acceleration range was ±8 G, the angular velocity range was ±1.000 dps, the sampling interval was 10 ms, and the average number of samples taken was 1. The tilt angles in the sagittal, frontal, and horizontal planes of the sacrum and femoral region during PHE were measured. PHE was an active movement, and the starting position of the PHE was in the supine position with hip extension at 0°. Subsequently, the patients were instructed to perform the right hip extension movement with the knee joint extended from the starting position (Figure 1). PHE was performed twice: the first time with a natural PHE (NPHE) and the second time with a modified PHE (MPHE), in which the participants were instructed to control their pelvic and, therefore, lumbar movements as much as possible. The motion angle was measured when the femoral inertial sensor was tilted at an angle of 10° in the direction of the hip extension on the sagittal plane (10° tilt) and at the maximum tilt. The tilt angle of the pelvis was defined as the angle of movement of the lumbar spine. In addition, the difference between the tilt angle of the femoral region and the pelvis was defined as the angle of movement of the hip joint.

### 2.3. Evaluation of Spinal Curvatures 

The alignment of the spine was evaluated using a spine analyzer (Spinalmouth, Index Ltd., Tokyo, Japan). The reliability of spine alignment measurements with a spine shape analyzer is good for both intra-rater reliability and inter-rater reliability [22,23,24]. The spinal curvatures were measured in the standing position, with the sensor on the spinous process from the seventh cervical vertebra to the third sacral vertebra, moving from the head to the caudal direction. The thoracic kyphosis angle was the sum of the angles between the upper and lower vertebrae from the 1st to the 12th thoracic vertebrae. The lumbar lordosis angle was defined as the sum of the angles between the upper and lower vertebrae from the first lumbar vertebra to the first sacral vertebra.

### 2.4. Statistical Analysis 

Statistical analysis was performed using SPSS, version 24 (IBM SPSS Statistics, Japan IBM, Tokyo, Japan). For the comparison of NPHE and MPHE angles, normality was confirmed by the Shapiro-Wilk test. Consequently, the paired t-test was applied. To understand the movement patterns of the lumbar and hip joints, the movement ratios of the lumbar and hip joints (lumbar/hip joint) were calculated for the NPHE and MPHE. In addition, we calculated the rate of change between the NPHE and MPHE (MPHE/NPHE) of the motion ratio of the lumbar and hip joints and used these values as an index of the motor control ability of the lumbar region. Pearson’s correlation coefficient was applied to the relationship between motor control ability and spinal alignment. The level of significance was set at 0.05, and all values are presented as the mean ± standard error. 

## 3. Results

### 3.1. Lumbar and Hip Joint Angles During PHE

Table 1 shows the lumbar and hip joint angles at a 10° tilt in PHE, and Table 2 shows the lumbar and hip joint angles at the maximum tilt. In the sagittal plane, the positive direction is described as the extension direction. In the frontal plane, the positive direction is described as the right pelvic elevation for the pelvis and the abduction direction for the hip joint. The positive direction of the horizontal plane is described as the posterior rotation of the pelvis and the external rotation direction for the hip joint.

In the sagittal plane, the lumbar angle was significantly greater for NPHEs than for MPHEs at a 10° tilt. In the horizontal plane, the angle of the lumbar spine was significantly smaller in the NPHE than in the MPHE. Conversely, in the sagittal plane, the hip joint angle was significantly smaller than that of the MPHE at a 10° tilt. Hip angles in the horizontal plane were significantly smaller for MPHE than for NPHE. In the frontal plane, only the hip joint angle showed significantly greater NPHE compared to MPHE.

In the sagittal plane, the lumbar angle was significantly greater for NPHEs than for MPHEs at the maximum tilt. In the horizontal plane, the lumbar angle was significantly smaller in the NPHE than in the MPHE. The hip angle was significantly greater in the frontal plane only in the NPHE group than in the MPHE group. In addition, the tilt angle of the femoral region sensor was investigated because the femoral elevation angle was different between NPHE and MPHE. As a result, the femoral elevation angle was significantly greater in the sagittal and frontal planes only for NPHE compared to MPHE.

### 3.2. Movement Patterns of the Lumbar and Hip Joints During PHE

The lumbar and hip joint movement ratios (lumbar/hip joint) of the PHE are shown in Table 3. There was a significant difference between the NPHEs and MPHEs in the sagittal plane only at the maximum tilt.

### 3.3. Joint Angles, Movement Patterns, Motor Control Ability of the Lumbar and Hip Joints During PHE, and Spinal Curvatures

The rate of change in NPHE and MPHE during PHE (MPHE/NPHE) is shown in Table 4. In addition, the thoracic kyphosis angle and lumbar lordosis angle are shown in Table 5. A positive value indicates flexion (kyphosis) of the spine, and a negative value indicates extension (lordosis) of the spine. The lumbar angle of the NPHE in the sagittal plane at maximum tilt was significantly correlated with the thoracic kyphosis angle (*p* = 0.05, *r* = 0.41). However, there were no significant correlations between the other lumbar and hip joint angles, lumbar/hip joint movement ratios, and rates of PHE changes with the thoracic kyphosis angle and lumbar lordosis angle. 

## 4. Discussion

This study aimed to clarify the relationship between lumbar and hip joint movement patterns, lumbar movement control ability during PHE, and spinal curvatures. The results of this study provide a basis for evaluating the movement pattern, motor control ability, and spinal alignment of the lumbar spine and may contribute to the prevention and intervention of low back pain. The results showed that the NPHEs had greater extensional movement of the lumbar region and lesser extensional movement of the hip joint than the MPHEs at 10° tilt. Even in the horizontal plane, the NPHE showed greater right pelvic posterior rotation and less external rotational movement of the hip joint than MPHE. Therefore, the extension and rotation of the lumbar spine appeared earlier in the NPHE than in the MPHE. Oh et al. [25] reported that the muscle activity of the erector spinae decreased and that the anterior tilt angle of the pelvis decreased with the abdominal drawing-in-maneuver motor control of the lumbar using the pressure biofeedback unit during PHE. In the present study, it is suggested that a similar effect was obtained by controlling the movement of the lumbar region by oral instructions. Tateuchi et al. [26] reported that the activity balance of the peri-hip muscles is related to pelvic movements during hip extension. In particular, they reported an association between increased muscle activity of the tensor fasciae latae muscle, decreased activity of the gluteus maximus muscle, and a delay in the timing of the onset of activity of the trunk muscles. Although this study did not evaluate muscle activity, it is not clear. However, it is possible that the differences in trunk and peri-hip muscle activity between NPHE and MPHE reduced lumbar movement. At maximum tilt, only the lumbar angle was significantly different in the sagittal and horizontal planes. Furthermore, at the maximum tilt in the sagittal plane, the tilt angle of the femur was significantly higher for the NPHE than for the MPHE. These results suggest that, to raise the lower limbs in the end range of NPHE, the femur raising angle is secured by additional lumbar movement than the MPHE instead of the hip joint.

From the results of this study, there was no relationship between the thoracic kyphosis angle and lumbar lordosis angle in the standing position and the ratio of motion of the lumbar and hip joints or the motor control ability of the lumbar during PHE. The results suggest that the lumbar and hip joint movement patterns and the ability to control lumbar movements in PHE are not associated with static spinal alignment. Therefore, when evaluating the lumbar region, it is considered that a comprehensive evaluation is required, not only one of the spinal alignments and the dynamic evaluation such as movement pattern and motor control ability. On the other hand, there was a correlation between the thoracic kyphosis angle and the angle of the lumbar spine at the maximum tilt of the NPHE. The thoracic spine is an area prone to decreased mobility [27], and the thoracic kyphosis angle is used as an indicator of thoracic spine extension limitation. An increased thoracic kyphosis is a risk factor for low back pain [28]. In addition, it is expected that the limitation of the thoracic spine in the direction of extension causes excessive movement of the lumbar spine in daily life. However, it is unclear whether lumbar hypermobility caused an increase in the thoracic kyphosis or whether the increase in thoracic kyphosis caused the hypermobility of the lumbar spine. 

One of the limitations of this study is that, although we were careful to avoid any misalignment during the measurement, the angle measured may differ from the true angle because of the misalignment caused by clothing and bands that hold the sensor in place. Furthermore, we did not measure the passive range of motion in the extension of the hip joint; therefore, it is not clear how much the patient moved with active movement in relation to the range of motion. Since there were only 14 participants, further studies are needed with more participants to verify the study findings. In addition, the participants of this study were healthy elderly people, and there is a possibility that the relationship between spinal alignment and movement patterns and motor control of the lumbar and hip joints may show different results for people with low back pain. Therefore, it is necessary to investigate the relationship between spinal alignment and movement patterns and motor control of the lumbar and hip joints during PHE in patients with low back pain and to clarify the factors and cutoff values associated with low back pain. Furthermore, because of the large individual differences in the movement patterns of the lumbar and hip joints in this study, it is necessary to investigate the relationship between each factor after classifying the participants. In this study, we mainly focused on the relationship between lumbar movement in the direction of extension and spinal alignment. Therefore, the standing position was chosen for the evaluation of spinal alignment. Different results may be obtained for the relationship between the flexional movement of the spine and spinal alignment in other postures, such as sitting. In addition, since the results of the present study were only for PHE movements from the lower limbs in the direction of spine extension, other movements such as trunk back-bending in the standing position need to be verified as well. 

## 5. Conclusions

There was no correlation between the spinal curvatures in the standing position, lumbar and hip joint movement patterns, and motor control ability during PHE. Therefore, during the evaluation and treatment of the lumbar region, it is necessary to take comprehensive decisions rather than only interpreting either static or dynamic evaluations.

## Figures and Tables

**Table 1:** Lumbar and hip joint angles at 10° tilt during PHE.

Directions	Lumbar Angle (°)			Hip Joint Angle (°)		
	Sagittal Plane	Frontal Plane	Horizontal Plane	Sagittal Plane	Frontal Plane	Horizontal Plane
NPHE	4.66 ± 0.17	−1.17 ± 0.08	−2.36 ± 0.19	5.68 ± 0.17	2.47 ± 0.23	−5.15 ± 0.59
MPHE	2.97 ± 0.19 *	−0.86 ± 0.06	−0.46 ± 0.18 *	7.39 ± 0.21 *	1.5 ± 0.26 *	−5.43 ± 0.53 *
95% confidence interval	0.89–2.49	−1.10–0.48	−2.91–−0.88	−2.61–−0.80	−0.33–2.28	−3.38–3.94

Data are presented as the mean ± standard error or minimum and maximum values. NPHE, Prone hip extension in a natural manner; MPHE, Modified prone hip extension with controlled lumbar movement. * Significant differences between NPHE and MPHE (*p* < 0.05).

**Table 2:** Lumbar and hip joint angle at the maximum tilt during PHE.

Directions	Lumbar Angle (°)			Hip Joint Angle (°)			Femoral Elevation Angle (°)		
	Sagittal Plane	Frontal Plane	Horizontal Plane	Sagittal Plane	Frontal Plane	Horizontal Plane	Sagittal Plane	Frontal Plane	Horizontal Plane
NPHE	16.06 ± 0.38	−1.68 ± 0.11	−11.97 ± 0.28	10.54 ± 0.42	9.42 ± 0.42	−10.14 ± 0.57	25.12 ± 0.60	−17.00 ± 1.02	6.46 ± 0.37
MPHE	10.09 ± 0.31 *	−1.26 ± 0.09	−5.20 ± 0.28 *	11.18 ± 0.45	5.88 ± 0.34 *	−7.56 ± 0.61	19.93 ± 0.47 *	−11.55 ± 0.68 *	3.89 ± 0.31
95% confidence interval	3.12–8.83	−1.48–0.63	−9.56–−3.97	−2.35–1.07	1.05–6.04	−7.02–1.87	0.30–10.06	0.16–4.97	−13.05–2.15

Data are presented as the mean ± standard error or minimum and maximum values. NPHE, Prone hip extension in a natural manner; MPHE, Modified prone hip extension with controlled lumbar movement. * Significant differences between NPHE and MPHE (*p* < 0.05).

**Table 3:** Lumbar and hip joint movement ratio (lumbar/hip joint) (%) during PHE.

Directions	10° Tilt			Maximum Tilt		
	Sagittal Plane	Frontal Plane	Horizontal Plane	Sagittal Plane	Frontal Plane	Horizontal Plane
NPHE	1.19 ± 0.27	−0.23 ± 0.27	0.22 ± 0.13	2.68 ± 0.86	−0.40 ± 0.13	0.74 ± 0.30
MPHE	1.04 ± 0.61	−0.17 ± 0.22	0.26 ± 0.17	1.33 ± 0.30 *	−0.39 ± 0.17	−1.83 ± 2.91
95% confidence interval	−0.78–1.08	−0.76–0.63	−0.52–0.45	0.05–2.64	−0.41–0.39	−3.69–8.83

Data are presented as the mean ± standard error or minimum and maximum values. NPHE, Prone hip extension in a natural manner; MPHE, Modified prone hip extension with controlled lumbar movement. * Significant differences between NPHE and MPHE (*p* < 0.05).

**Table 4:** Rate of change in NPHE and MPHE during PHE (MPHE/NPHE).

Directions	Sagittal Plane	Frontal Plane	Horizontal Plane
10° tilt	2.60 ± 0.76	−3.76 ± 3.51	0.25 ± 0.78
Maximum tilt	1.94 ± 0.15	3.03 ± 2.04	1.27 ± 1.31

Data are presented as mean ± standard error. NPHE, Prone hip extension in a natural manner; MPHE, Modified prone hip extension with controlled lumbar movement.

**Table 5:** Thoracic kyphosis angle and lumbar lordosis angle.

Thoracic Kyphosis Angle	Lumbar Lordosis Angle
32.9 ± 2.6	−8.0 ± 2.7

Data are presented as mean ± standard error. A positive value indicates flexion (kyphosis) of the spine, and a negative value indicates extension (lordosis) of the spine.
